# Enhancement of the structure and linear/nonlinear optical properties of PVA/chitosan/Ag nanocomposites for optoelectronic and antibacterial applications

**DOI:** 10.1038/s41598-025-12029-z

**Published:** 2025-07-26

**Authors:** H. Elhendawi, R. M. Ramadan, M. S. Abdel-Aziz, T. Fahmy, A. M. Ali

**Affiliations:** 1 Basic Sci. Dept., Faculty of Eng., Delta University for Science and Technology, Gamasa, Egypt; 2 Microwave Physics and Dielectrics Dept., Physics Research Institute, National Research Centre (NRC), Dokki, Giza, 12622 Egypt; 3 Microbial Chemistry Dept., Biotechnology Research Institute, National Research Centre (NRC), 33 El-Bohouth St.Dokki, Giza, 12622 Egypt; 4 Polymer Research Group, Physics Dept., Faculty of Sci., Mansoura Uni., Mansoura, 35516 Egypt; 5 Physics Dept., Faculty of Sci., Mansoura Uni., Mansoura, 35516 Egypt

**Keywords:** PVA, Chitosan, AgNPs, Dislocation density, Photoconductivity, Antibacterial, Biophysics, Materials science

## Abstract

Polyvinyl alcohol/chitosan/silver (PVA/Cs/Ag) nanocomposites are prepared using casting method for eco-friendly applications. The microstructure and optical properties are investigated using X-ray diffraction, Fourier transform infrared and UV/Vis spectroscopy. XRD analysis revealed that the structural parameters such as lattice strain, crystallites per unit area, dislocation density and stacking fault are affected upon incorporating AgNPs. UV/Vis measurements exhibited that the indirect and direct optical bandgap are reduced from 4.34/5.22 eV for PVA/Cs blend to 3.38/4.78 eV for PVA/Cs/1wt%Ag nanocomposite. Analysis of Wemple-DiDomenico model exhibited clear enrichment of the linear/nonlinear parameters and it is found that the third order nonlinear optical parameter (χ^(3)^) and nonlinear refractive index, n_2_ are enhanced and increased from (0.025 × 10^–12^/0.612 × 10^−12^ esu) for PVA/Cs blend to (4.874 × 10^–12^/2.413 × 10^–12^ esu) for PVA/Cs/1wt%Ag nanocomposite. The significant decrease in the energy gap as well as the remarkable change in the nonlinear optical parameters make these materials a strong candidate for many applications in the optoelectronic field. Antibacterial activity measurements strongly suggest that these nanocomposites could be used in the fabrication of antibacterial devices with biomedical applications.

## Introduction

Polyvinyl alcohol (PVA) is one of the most interesting and studied polymers due to its diverse and unique properties, making it an excellent organic material for a variety of applications. PVA is water-soluble polymer with biodegradability, excellent biocompatibility, good adhesive properties and thermal stability. These properties make PVA and its composites are useful for a range of technologies, such as biomedical materials, ultrafiltration, ion exchange membrane and protective coatings^1,2^. Chitosan (Cs) has been widely studied as a natural cationic biopolymer among the natural polymers. Cs has biodegradability, excellent biocompatibility, non-toxicity, bioactivity, artificial skin and bone substitutes and also the solubility in aqueous medium for packaging applications^3,4^. Blending two or more polymers for producing novel polymeric hosts with specific properties is a new scientific trend for updated applications whereas polymeric nanocomposites are obtained by doping small amounts of nano-fillers into the matrix of the polymer blend. PVA and chitosan can be mixed to prepare a new polymeric matrix for many applications in different fields. The attached hydroxyl groups to the main-chain of PVA act as a source of hydrogen bonding for improving the complexation process and facilitating the blending with the polymers^5,6^. On the other hand, Cs as a derivative of chitin consisting of 2-amino-2-deoxy-d-glucose and 2-acetamido-2-deoxy-D-glucose is the most abundant polymer in nature and plays an important role in medical field due to its biocompatibility, antimicrobial as well as antifungal activities^7^. The hydroxyl (–OH) and amine (–NH_2_) groups in the chitosan main-chain are enhancing the complexation process with the inorganic fillers^8^. Blending of PVA and Cs will produce a polymer blend matrix as a new host for different types of doping materials.

Metal nanoparticles (MNPs) due to their surface plasmon resonance (SPR) can exhibit exceptional and tunable optical properties. Coherent collective excitations of the free electrons of these particles makes the metal nanoparticles have high absorption as well as sub-wavelength photonics and light scattering capacity^9^. SPR in metal nanoparticles is important for various applications such as, near-field optical microscopy, molecular sensing and light focusing^10^. Moreover, the phenomena of SPR are significant for the nonlinear optical susceptibility. Consequently, the plasmonic material is unique for use in the design of optoelectronic devices such as the ultrafast optical switches.

Metal-polymer nanocomposites (MPNCs) are receiving great attention because the properties of nanocomposites differ from those of polymers due to the interfacial interactions between large polymer molecules and metal nanoparticles. Hence, MPNCs have an important role in many applications such as, shielding, industrial, medical and biological fields^11,12^. In particular, the incorporation of silver nanoparticles (AgNPs) within the polymer matrix exhibits a significant impact on the structural, optical and electrical properties. Therefore, it can be adapted for various applications such as microchips, photonics, charge storage capacitors and catalysis^13^. The polymeric nanocomposites with great dielectric properties are highly desirable for the design of supercapacitors and electronic devices. As AgNPs have a large surface area they are used in medical products, which enhances contact with microorganisms making them effectively antimicrobial^14–17^.

Our objective in this study is to improve the optical properties of PVA/Cs polymer blend including bandgap, dispersion energy and linear/nonlinear parameters by doping with AgNPs. The relatively small optical band gap value (2.51 eV) of AgNPs can play an important role as filler materials for diverse optoelectronic applications such as energy storage, solar cells, and LEDs. The microstructure modification of PVA/Cs/Ag nanocomposites is investigated by X-ray diffraction and FT-IR spectroscopy. Linear/nonlinear optical parameters are estimated based on the application of Wemple-DiDomenico model. The results obtained revealed the suitability of PVA/Cs/Ag nanocomposites for various optoelectronic applications. The antibacterial activity has also been studied due to the high activity of AgNPs which qualifies the PVA/Cs/AgNPs composites for many daily medical applications.

## Experimental procedure

### Materials

Chitosan powder with a molecular weight of ∼300,000 and N-deacetylation degree of 86% is purchased from Acros Organics (USA). PVA (C_2_H_4_O)_n_ supplied by laboratory Rasayan (India) with a molecular weight of ∼16,000. Silver nitrate is purchased from SRL (India) with a molecular weight of 169.87.

### Preparation of PVA/Cs blend

1% chitosan (w/v) is dissolved in distilled water with 2% acetic acid (v/v) at 313 K for 5 h using a magnetic stirrer to obtain a completely transparent solution. 1% polyvinyl alcohol (w/v) was dissolved in distilled water at 353 K for 6 h to obtain a clear solution. This ratio of Cs and PVA is chosen to ensure a homogeneous and compatible mixture. The two solutions were then mixed together under continuous mechanical stirring for 1 h to obtain a homogeneous mixture. The mixture is then poured into glass dishes in an oven at 333 K for 48 h to obtain PVA/Cs polymer blend films.

### Preparation of PVA/Cs/silver-based nanoparticle films

**Fig. 1:**
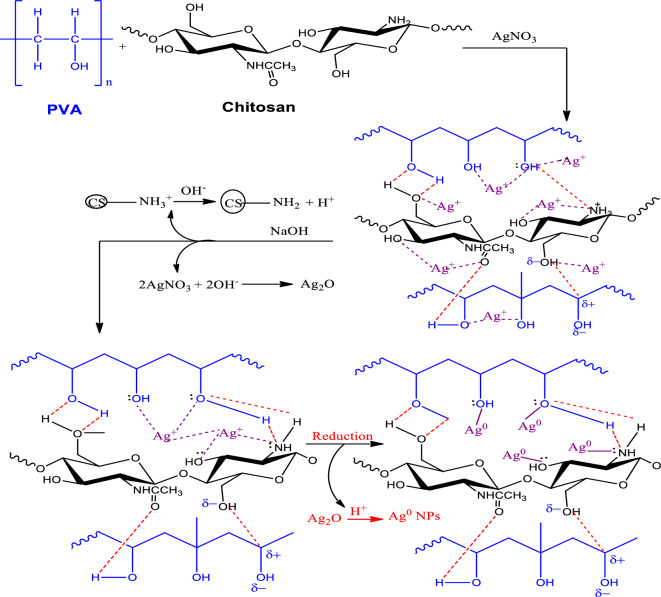
The interaction mechanism between PVA/Cs blend and AgNO_3_.

A small amount of silver nitrate diluted in distilled water is added to the PVA/Cs solution. Silver concentrations of 0.1, 0.5 and 1wt% (g/100g chitosan) are simulated by preparing several PVA/Cs solutions with different concentrations of silver nitrate. The mixture is shaken until completely dissolved while protected from light. A casting method is used to prepare the films, which are then dried for 2 days at 333 K. The PVA/Cs films are immersed in a 0.1 M NaOH solution for 20 h at room temperature and protected from light. The films are neutralized by NaOH to make them insoluble in water at a pH higher than the pKa of chitosan, and the hydroxide ions increased the reducing power of chitosan, which accelerated the reduction reaction of silver ion and formation of silver-based nanoparticles. After neutralization, the films are washed with distilled water and dried at 313 K in an oven for 2 days. The proposed interaction between the PVA/Cs blend and silver nitrate is depicted in Fig. 1.

### Characterization techniques

#### XRD

X-ray diffraction patterns are measured for all samples using Philips PW 1390 X-ray diffractometer with a monochromator beam of Cu K_α_ radiation at λ = 1.5406 Å (V = 40 kV and I = 40 mA). The 2θ angle is scanned in the range of 4° to 70° at speed of 2θ = 2°/min.

#### Fourier transformation infrared measurement (FTIR)

The Fourier transformation infrared spectra are measured in the wavenumber range 4000–400 cm^−1^ for all samples at room temperature using (IR) Spectrometer (MATTSON 5000 FTIR).

#### UV/Vis spectroscopy

UV/Vis measurements are performed in the range from 200 to 1100 nm by UV–Vis spectrophotometer (UV-1601 PC, Shimadzu, Japan).

### Antibacterial activity

#### Disc agar diffusion method (sensitivity test)

The antimicrobial activities of PVA, Cs, C, D, E and F are studied using agar diffusion method. *Staphylococcus aureus* (*S. aureus*) ATCC 6538-P and *Escherichia coli* (*E. coli*) ATCC 25933 are used as G+ ve and G−ve bacterial test strains whereas *Candida albicans* (*C. albicans*) ATCC 10231and *Aspergillus niger* (*A. niger*) NRRL-A326 are considered as fungal test microbes. Nutrient agar plates are heavily inoculated regularly with 0.1ml of 10^5^–10^6^ cells/ml in case of bacteria and yeast whereas potato dextrose agar medium is used in case of *A. niger*. Samples are placed on inoculated plates. To allow maximum diffusion, the plates are kept incubated for 2 to 4 h at 4 °C. The plates are then incubated at 37 °C for 1 day for bacteria and at 30 °C for 2 days in an upright position for allowing maximum growth of the organisms. The antimicrobial activity of the test agent is calculated by estimating the inhibition zone diameter in millimeters. The experiment is performed several times and the average reading is taken.

#### Colony forming unit (CFU) method

Colony forming unit method is carried out accurately for determining the antibacterial activity of the prepared samples. *S. aureus* and *E. coli* are selected as test strains. Nutrient broth medium is separately inoculated with both test microbes as controls. Samples (250 mg/10 ml culture medium) are also added to this medium and inoculated with test strains. After 2 days incubation at 37 °C, a serial dilution from the samples containing culture and the controls has been done (10^–1^–10^–4^). Microbial inhibition is estimated the colony forming units (CFU) using inoculating the petri-dishes containing solidified nutrient agar medium with 50 µl from each dilution and evaluating the rate of reduction growth (R) of the treated samples in relation to control (untreated sample) according to the following equation^18^:

$$R(\% ) = \,\,\frac{B\, - \,A}{B}\,x\,100$$

where *A* is CFU/ml of the treated sample after 16 h of incubation and *B* is CFU/ml of the untreated sample after the same period of incubation time.

## Results and discussion

### XRD

**Fig. 2:**
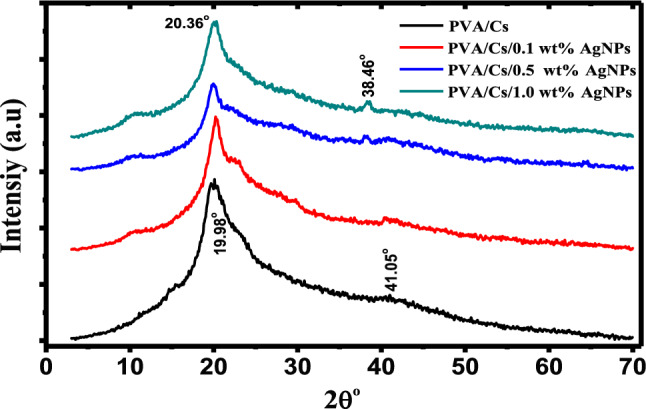
XRD of PVA/Cs and PVA/Cs/Ag nanocomposite samples.

X-ray diffraction pattern investigation is a reliable technique to analyze the crystal structure of polymeric composites. It is confirmed that the incorporation of nano-fillers into the polymer matrix enhanced the amorphous phase through hydrogen bonds disruption and led to the creation of an amorphous structure. Figure 2 displays XRD patterns of PVA/Cs/Ag nanocomposite samples in the range from 2θ = 4° to 70°. It is observed that XRD pattern of PVA/Cs blend is characterized by a diffraction peak at 19.98°. This peak indicated to the PVA/Cs semicrystalline nature due to the intra/intermolecular interaction via hydrogen bonding between PVA/Cs chains^19^.

On the other hand, the incorporation of AgNPs exhibited a diffraction peak at 38.46° corresponding to (111) plane of silver and the main diffraction peak of PVA/Cs is shifted to 20.36° and its intensity is decreased upon increasing AgNPs content. The decrease in intensity indicates an increase in the degree of amorphousness of the nanocomposite samples. This is due to the interaction between the PVA/Cs matrix and AgNPs, which results in a change in the structure of the blend with the variation in the content of silver nanoparticles^20^. Also, the shift in the peak position can be attributed to the variation in the electrostatic interactions among AgNPs and chains of PVA/Cs matrix which will alter the structure of the blend with changing the content of AgNPs, leading to an increase in the amorphous regions in the samples^21^. Similar behavior is published previously for various polymeric nanocomposites^22,23^. Crystallite size (*D*) and internal lattice strain (ε) values of our investigated samples are evaluated by Debye–Scherrer equations, as follow^24,25^:

1 $$D = \,0.94\,\lambda /\beta \,\cos \theta$$

2 $$\varepsilon \, = \,\beta /4\,\tan \theta$$

3 $$R = \,\frac{5\lambda }{{\,8\sin \theta }}$$

where *λ*, *β* and *θ* are the wavelength of the X-ray, FWHM in radians and the diffraction angle of the peak, respectively. Various other structural parameters such as, stacking fault (*SF*), dislocation density (δ) and the number of crystallites per unit area (*N*_*c*_) are computed using the following equations^26^:

4 $$SF = \beta \,\left[ {\frac{2\pi }{{45\,(\tan \,\theta )^{0.5} }}} \right]\,$$

5 $$\delta = \,\frac{1}{{\,D^{2} }}$$

6 $$N{}_{c} = \,\frac{d}{{\,D^{3} }}$$

where *d* is the sample thickness. The parameters of the structure are calculated and given in Table 1.

**Table 1:** Structural parameters of PVA/Cs/Ag nanocomposites.

Structural parameters	PVA/Cs	PVA/Cs/0.1 wt%AgNPs	PVA/Cs/0.5 wt%AgNPs	PVA/Cs/1.0 wt%AgNPs
*D* (nm)	6.19	7.88	7.14	5.54
ε	0.034	0.026	0.029	0.037
R(Å)	5.55	5.47	5.55	5.44
*SF*	0.066	0.041	0.055	0.069
δ (nm^−2^)	0.030	0.012	0.021	0.033
*N*_*c*_ (nm^−2^)	300.9	93.63	281.7	321.8

### FTIR spectroscopy

**Fig. 3:**
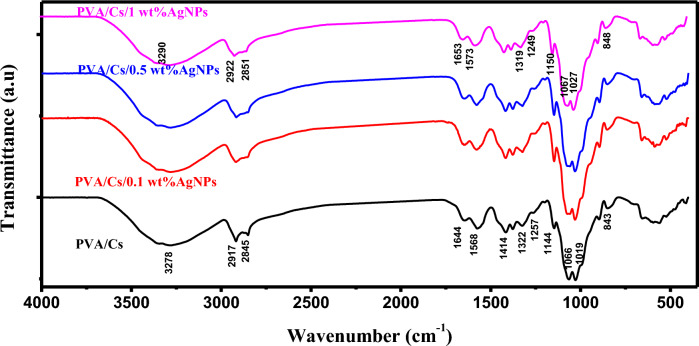
FTIR spectra of PVA/Cs/Ag nanocomposite samples.

The FTIR spectra of PVA/Cs and PVA/Cs/Ag nanocomposites are presented in Fig. 3. The characteristic bands of PVA and Cs are displayed in the FTIR spectrum of PVA/Cs blend. The broad band centered at 3278 cm^−1^ is assigned to OH/NH groups interact with the oxygen of C=O groups. The bands at 2917 cm^−1^ and 2845 cm^−1^ are attributed to CH stretching vibration^27,28^. The characteristic bands of amide I and amide II are observed at 1644 cm^−1^ and 1568 cm^−1^ are ascribed to C=O stretching vibration and to N–H deformation, respectively^29^. The bands at 1144, 1066 and 1019 cm^−1^ are attributed to glycosidic linkage and glucose ring vibration of C–O–C^30^. The bands at 1414, 1322, 1257 and 843 cm^−1^ of PVA are attributed to –CH_2_ bending, C–H deformation, OH bending, C–H wagging and C–H rocking, respectively^31^.

FTIR spectra of PVA/Cs/AgNPs composites showed an irregular shift in the position of the main characteristic bands. The position of O–H/N–H band is shifted towards high wavenumber to appear at 3290 cm^−1^ with shift in the order of 12 cm^−1^ (blue shift), indicating that there is a cross-linking reaction between hydroxyl groups of PVA/Cs matrix and Ag nanoparticles^32^. Also, bands of CH stretching of PVA/Cs blend are found to be blue shifted with shift orders of 5 and 6 cm^−1^ and observed at 2922 and 2851 cm^−1^ in PVA/Cs/1 wt% AgNPs. Moreover, the bands of amide I and amide II are blue shifted with shift orders of 9 and 5 cm^−1^ with increasing the content of AgNPs and appeared at 1653 and 1573 cm^−1^. The characteristic bands of PVA are found to be blue and red shifted as illustrated in the FTIR spectrum of PVA/Cs/1 wt% AgNPs. These observations are due to the strong interaction among the chains of PVA/Cs and Ag nanoparticles by making co-ordinations between the functional groups of PVA/Cs blend and AgNPs. This indicates the formation of hydrogen bonding between the PVA/Cs blend and AgNPs. Hence, it can be said that the Ag nanoparticles are located between chains of PVA/Cs linked via functional groups^33^.

### UV/Vis spectroscopy

**Fig. 4:**
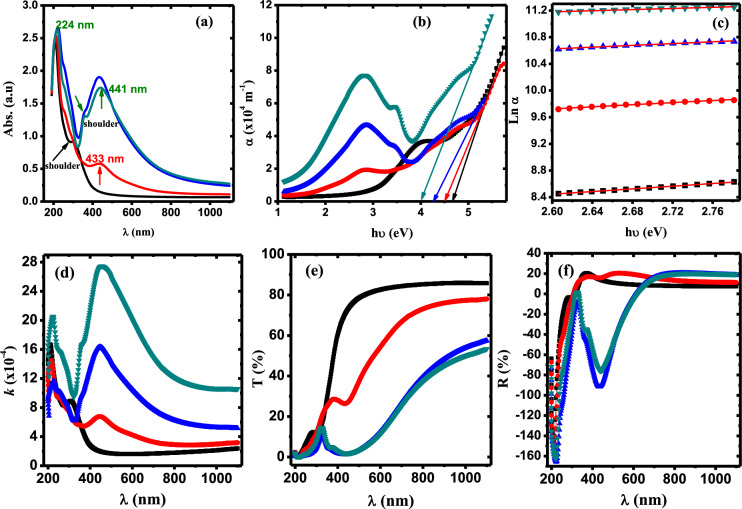
(**a**) Absorbance against λ, (**b**) α against hυ (**c**) Ln α against hυ, (**d**) *k* against *λ*, (**e**) T(%) against *λ* and (**f**) R(%) against *λ* for PVA and PVA/Cs/Ag nanocomposites. (Filled black square) PVC/Cs, (filled red circle) PVC/Cs/0.1 wt% AgNPs, (filled blue triangle) PVC/Cs/0.5 wt% AgNPs (filled green inverted triangle) PVC/Cs/1 wt% AgNPs.

The impact of AgNPs on the optical properties of PVA/Cs is explored through measurements of the UV/Vis spectroscopy. Figure 4a depicts UV–Vis spectra of PVA/Cs/Ag nanocomposites in the wavelength range of 200–1100 nm. It is found that the absorption spectrum of PVA/Cs blend displayed an absorption peak at 210 nm and a shoulder in the range 277–306 nm. These transitions are ascribed to π–π* and n–π* transitions due to the existence of C=O of the carbonyl group^34^. The absorption bands of pure PVA/Cs blend are shifted to appear at 224 nm and also in the range of 350–370 nm in the spectra of PVA/Cs/Ag nanocomposites. Moreover, the spectra of nanocomposite samples exhibited an interesting absorption band at 433 nm in PVA/Cs/0.1 wt%Ag spectrum and shifted to 441 nm in the PVA/Cs/1 wt%Ag nanocomposite spectrum. This band indicated that AgNPs are existed in the nanocomposites and is attributed to the surface plasmon resonance (SPR) properties of silver nanoparticles^15^.

Absorption spectra of PVA/Cs/Ag nanocomposites displayed a noticeable increase in the absorbance followed by a red shift of SPR peak to the high wavelength from 433 to 441 nm. This shift in the SPR band depends mainly on the particle shape and size^35^. It is previously published in other studies that the SPR peak shifts to higher wavelengths due to increased particle size resulting from particle aggregation^36^. Our results are in good agreement with what is previously published regarding the dependence of the SPR peak position on the size of silver nanoparticles^37,38^. Amirjani et al. reported that a red shift in the position of SPR peak from 390 to 460 nm with variation in the size of Ag nanoparticles^37^. Alzoubi et al.reported that shift in SPR peak position depends mainly on the size of NPs and their shape^38^. The broadening of SPR band indicates that the AgNPs are polydispersed in the matrix of PVA/Cs blend. This band is ascribed to the spherical colloidal AgNPs absorption because of excitations of surface plasmon vibration^35^. It is observed that the higher the content of AgNPs, the greater the intensity of absorption, which enhanced the surface area due to the nano-size of Ag, and thus strong absorption is obtained^36^. Consequently, as a result of the above results, these nanocomposites can be used in the applications of UV shielding.

Figure 4b displays the dependence of absorption coefficient, α (α = 2.303 *A/d*, where, *A* and *d* are the absorbance and sample thickness, respectively) on the photon energy (*hυ*) for PVA/Cs/Ag nanocomposites. The behavior of the absorption coefficient exhibited a linear relation at higher values of *hυ*. The intercepts of extrapolation of the linear portion to zero absorption coefficient (α = o) with the axis of photon energy are taken as the absorption edge energy values and summarized in Tables 2, 3. The absorption coefficient depends exponentially on the photon energy and can be expressed using Urbach relation as follows^39^:

7 $$\alpha \, = \,\alpha {}_{o}\,\exp \left( {{\raise0.7ex\hbox{${h\upsilon }$} \!\mathord{\left/ {\vphantom {{h\upsilon } {E_{U} }}}\right.\kern-0pt} \!\lower0.7ex\hbox{${E_{U} }$}}} \right)$$

where *α*_*o*_ is a constant and *E*_*U*_ is the Urbach tail energy. Variation of Ln α versus the photon energy is illustrated in Fig. 4c. The values of *E*_*U*_ are evaluated by knowing the slope values of the fitted lines in Fig. 4c and given in Tables 2, 3. It is found that *E*_*U*_ values are increased with increasing AgNPs content in the matrix of PVA/Cs. This means that the incorporation of AgNPs leads to redistribution in the energy states and thus a large number of possible transition from tail to tail and from bands to tail are allowed^40–42^. The increase in *E*_*U*_ values ​​confirms the increase in the material disorder, as previously indicated by the increase in the lattice strain values above ​​in the section of X-ray analysis. Also, density of defects and distorted bonds relaxation are known by estimating the steepness parameter (β) and the electron–phonon interactions strength (*E*_*e-p*_). β and *E*_*e-p*_ values are calculated as follow^43^:

8 $$\beta = {k_{B}T}/{E_{U}}, \quad E_{e - p} = 2/{3\beta}$$

where *k*_*B*_ and *T* are the Boltezman’s constant and room temperature, respectively. The calculated values of β and *E*_*e-p*_ are listed in Tables 2, 3. It is found that the β is decreased while *E*_*e-p*_ is increased upon increasing AgNPs content. This behavior indicates that the defect density states (DDS) near the absorption edge is increased, meaning that crystallinity of PVA/Cs blend is decreased with increasing Ag nanoparticles content^44^.

**Table 2:** The optical parameters of PVA/Cs/Ag nanocomposite samples.

Samples	Optical parameters						
	*E*_*ed*_* (eV)*	*E*_*U*_ (eV)	*β*	*E*_*e-p*_	*E*_*gi*_ (eV)	*E*_*gd*_ (eV)	*Ncc*
PVA/Cs	4.69	1.00	0.025	25.78	4.34	5.22	43
PVA/Cs/0.1wt%AgNPs	4.50	1.28	0.020	33.06	4.11	5.07	46
PVA/Cs/0.5wt%AgNPs	4.28	1.49	0.017	38.48	3.75	4.91	49
PVA/Cs/1.0wt%AgNPs	3.98	2.38	0.010	61.39	3.38	4.78	52

**Table 3:** Refractive index values according to the different models.

Sample	*E*_*ed*_* (eV)*	*n*_*RV*_	*n*_*M*_	*n*_*HV*_	*n*_*Re*_	*n*_*KS*_	*n*_*Average*_
PVA/Cs	5.22	0.843	2.006	1.605	1.939	1.976	1.674
PVA/Cs/0.1wt%AgNPs	5.07	0.936	2.020	1.614	1.968	1.995	1.706
PVA/Cs/0.5wt%AgNPs	4.91	1.035	2.036	1.623	2.000	2.015	1.742
PVA/Cs/1.0wt%AgNPs	4.78	1.116	2.049	1.631	2.027	2.033	1.771

The extinction coefficient (*k* = *λα/4π*) is an important optical parameter that describe changes in the absorption of the material after exposing to the electromagnetic waves. Figure 4d displays the extinction coefficient dependence of PVA/Cs/Ag nanocomposites on the wavelength. It is observed that *k* values are increased as the content of AgNPs in the PVA/Cs matrix increased. Since the extinction coefficient is closely related to the absorption coefficient, this behavior is attributed to an increase in the absorption coefficient (α). Moreover, the increase of AgNPs in the PVA/Cs matrix contributed to the increase of photon scattering in the higher wavelength region resulting in an increase of the extinction coefficient.

Figure 4e illustrates the variation of the transmittance (T%) of the pure PVA/Cs blend and PVA/Cs/Ag nanocomposite versus the wavelength. A sharp increase in the transmittance is observed in the UV region where *λ* < 400 nm, while in the visible region, at *λ* = 700 nm, the transmittance is found to decrease from 86.82% for pure PVA/Cs to become 23.82% in the PVA/Cs/ 1wt%Ag nanocomposites. Increasing the content of AgNPs in PVA/Cs matrix will lead to an increase in the number of scattered photons and, subsequently, a decrease in the optical transmittance in the PVA/Cs/Ag nanocomposites. Since the pure PVA/Cs matrix does not contain free electrons, it has a high optical transmittance. On the contrary to pure PVA/Cs, the decreasing of the optical transmittance in the PVA/Cs/Ag nanocomposites is attributed to formation of surface layer of covalent bonds between the PVA/Cs and Ag nanoparticles which, in turn, will increase the density of the samples and consequently will decrease transmission of the incident light. The electronic transition to the higher energy levels will contribute to filling some vacancies in the energy bands and thus, part of the incident light cannot pass through the nanocomposite samples. However, the transmittance values of PVA/Cs/Ag nanocomposites qualify them to be used for optical coatings purposes^45^. Figure 4f illustrates the dependence of reflectance on wavelength. It is observed that, as the AgNPs content increased in PVA/Cs matrix, the reflectance of the nanocomposite samples in the visible region is increased. Hence, we can say that the changes in the values of T and R reflected the effect of AgNPs on the electronic structure modification of the PVA/Cs matrix.

### Optical band gap

**Fig. 5:**
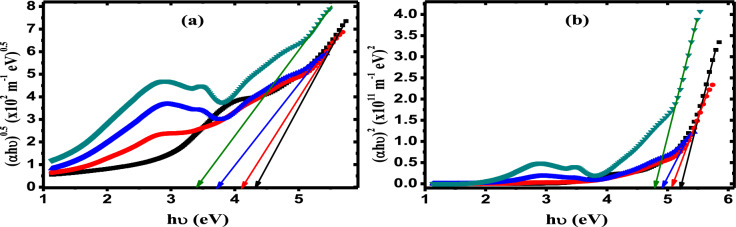
(**a**) (αhυ)^0.5^ and (**b**) (αhυ)^2^ against hυ. (Filled black square) PVC/Cs, (filled red circle) PVC/Cs/0.1 wt% AgNPs, (filled blue triangle) PVC/Cs/0.5 wt% AgNPs (filled green inverted triangle) PVC/Cs/1 wt% AgNPs.

The optical bandgap of PVA/Cs/Ag nanocomposites is evaluated using the following eqn.^46^:

9 $$(\alpha h\upsilon ) = \,C\,(h\upsilon \, - E_{g} )^{y}$$

where *C* is a constant and *y* is an exponent and its value depends on the transition type and equals to 1/2 and 2 for allowed direct and allowed indirect transitions, respectively. Figure 5 displays the dependence of (αhυ)^0.5^ and (αhυ)^2^ on hυ for PVA/Cs/Ag nanocomposites. The values of optical bandgap are evaluated by extrapolation the linear portion of (αhυ)^0.5^ and (αhυ)^2^ at their intersection with the x-axis of (hυ) at (αhυ)^0.5^ = 0 and (αhυ)^2^ = 0. The estimated values of indirect (*E*_*gi*_) and direct (*E*_*gd*_) bandgap are given in Table 2. It is observed that the values of (*E*_*gi*_) and (*E*_*gd*_) are decreased from 4.34 to 3.38 eV and from 5.22 to 4.78 eV, respectively. This reduction in both (*E*_*gi*_) and (*E*_*gd*_) values can be interpreted based on the fact that the incorporation of AgNPs will form charge-transfer complexes (CTCs) between AgNPs and OH/NH_2_ groups in the PVA/Cs matrix which leads to increased optical conductivity and variations in the values of *E*_*g*_ by giving additional charges to the PVA/Cs network^47^. The defects resulting from the presence of silver nanoparticles in the PVA/Cs matrix also contribute to the formation of localized states in the band gap, which facilitates the movement of electrons between these states. Thus, this increase in localized states leads to an increase in the degree of disorder in the matrix of PVA/Cs, as also proven by the increase in the *E*_*U*_ energy values ​​as mentioned above^48^. The carbon cluster (*N*_*cc*_) values of our investigated samples are calculated by the optical bandgap using the following equation^49^:

10 $$\sqrt {N_{cc} } = \,34.4\,E_{dg}^{ - 1}$$

It is evident that the *N*_*cc*_ value is enhanced and changed from 43 for PVA/Cs to 52 for PVA/Cs/1 wt% AgNPs. The enhancement of *N*_*cc*_ is due to the conjugation that occurred between the monomer units in the matrix of PVA/Cs blend after addition AgNPs.^6^. This enhancement of *N*_*cc*_ is also due to more defects that generated in the PVA/Cs matrix with increasing AgNPs content, which led to the creation of additional low-energy states, and then a decreasing in the optical bandgap values, which in turn led to the improvement of *N*_*cc*_ values.

### Energy gap-refractive index correlation

Optical parameters such as high and static-frequency dielectric constants and atomic polarization which would contribute significantly to the use of optical material in optoelectronics applications are correlated directly with the index of refraction (*n*). Moreover, the refraction index is related to the optical bandgap and the relationship between them is used in the designing of optoelectronic devices. The correlation between energy gap and refractive index is described by different models. Ravindra et al. suggested that the relation between *n* and *E*_*g*_ can be written as follow^50^:

11 $$n_{RV} = \,4.08 - \,0.62E_{g}$$

Since, the energy levels of the materials can be scaled by a factor of $\varepsilon_{opt}^{ - 2}$, where *ε*_*opt*_ = *n*^*2*^, Moss proposed the following eqn.^51^:

12 $$n_{M} = \,\left( {{\raise0.7ex\hbox{${95}$} \!\mathord{\left/ {\vphantom {{95} {E_{g} }}}\right.\kern-0pt} \!\lower0.7ex\hbox{${E_{g} }$}}} \right)^{0.25}$$

Hervé-Vandamme suggested the following equation based on the vibrations theory^52^:

13 $$n_{HV} = \,\left[ {1 + \left( {\frac{{E_{H} }}{{3.4 + E_{g} }}} \right)} \right]^{0.5}$$

where *E*_*H*_ = 13.6 eV and represents hydrogen ionization energy. Other relations are suggested and can be represented using the following equations^53,54^:

14 $$n_{{\text{Re}}} = \,\ln \,\left( {36.3\,/E_{g}^{{}} } \right)^{{}}$$

15 $$n_{KS} = \,3.366\,E_{g}^{ - 0.322}$$

Using these models, the values of *n* are estimated for PVA/Cs/Ag nanocomposites and summarized in Table 3. It is clear that the average value of *n* of PVA/Cs is changed from 1.674 to 1.771 for PVA/Cs/1 wt%Ag, indicating that AgNPs modified PVA/Cs matrix and produced a denser nanocomposites with high values of refractive index and low optical bandgap. The polymeric nanocomposites that characterized by high values of refractive index can be used in the solar cells and anti-reflective coatings fabrication. Thus they have promising applications in photonics field due to their ability to reduce the reflection loss at interfaces^55^.

### Refractive index analysis

The refractive index, *n* is an essential parameter for all the optical materials. It contributes significantly to the selection of materials used in optical applications or used in the manufacture of optical devices. Hence, the behavior of the refractive index must be studied, as many optical phenomena depend on the value of refractive index. The refractive index is closely related to the local field within the material as well as the electronic polarization of the ions. Refractive index depends mainly on the density of the materials, molecular weight and bonds strength and is correlated directly to the extinction coefficient (*k*) and reflectance (*R*) as follow^56^:

16 $$n\, = \,\left( {\frac{1 + R}{{1 - R}}} \right)\, + \,\sqrt {\frac{{\left( {1 + R} \right)^{2} }}{{\left( {1 - R} \right)^{2} }}\, - \,\left( {1 - k} \right)^{2} }$$

**Fig. 6:**
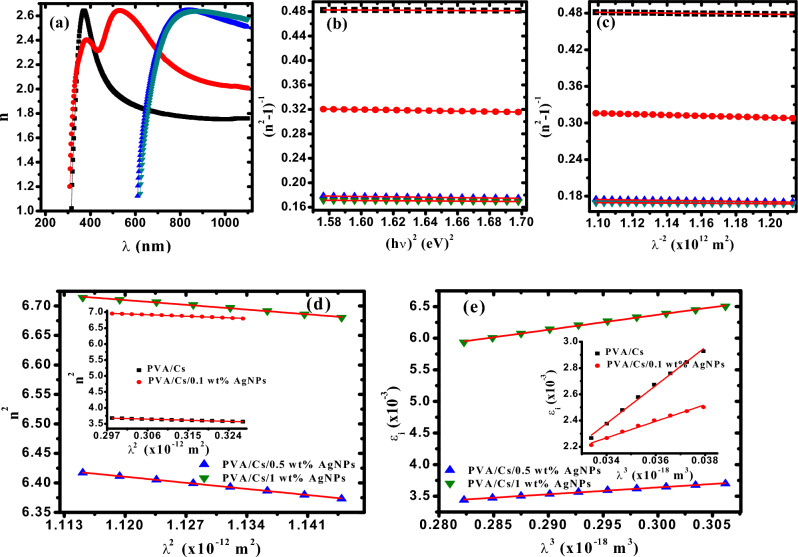
(**a**) n against *λ*, (**b**) (n^2^-1)^−1^ against (hυ)^2^, (**c**) (n^2^-1)^−1^ against λ^−2^, (**d**) n^2^ versus λ^2^ and (**e**) ε_i_ versus λ^3^. (Filled black square) PVC/Cs, (filled red circle) PVC/Cs/0.1 wt% AgNPs, (filled blue triangle) PVC/Cs/0.5 wt% AgNPs (filled green inverted triangle) PVC/Cs/1 wt% AgNPs.

Figure 6a shows the dependence of refractive index on the wavelength for PVA/Cs/Ag nanocomposites. It is observed that the refractive index is enhanced with increasing the content of AgNPs and changed from 1.79 for PVA/Cs blend to 2.68 for PVA/Cs/Ag nanocomposites at λ = 750 nm in the visible range. The apparent increase in refractive index results from the interaction of AgNPs with the PVA/Cs chains, leading to an increase in the density of the nanocomposite samples. Also the behavior of refractive index showed a normal dispersion with increasing the wavelength. Hence, the single oscillator model (SOM), which is introduced by Wemple and Di Domenico can be applied to interpret the refractive index dispersion^56^. The correlation between *n* and *hυ* below the interband absorption edge is expressed using as follows:

17 $$\left( {n^{2} - 1} \right)^{ - 1} \, = \,\frac{{E_{0} }}{{E_{d} }} - \,\frac{1}{{E_{d} E_{0} }}\,\,\left( {h\upsilon } \right)^{2}$$

where (*E*_*o*_) is the single oscillator energy and measures the electronic transition intensity and (*E*_*d*_) represents the dispersion energy which is a measure of the strength of the interband optical transition. Figure 6b demonstrates the variation of (*n*^*2*^*-*1)^−1^ versus (*hυ*)^2^ and by knowing the intercept (*E*_*0*_*/E*_*d*_) and slope (-1/*E*_*d*_*E*_*0*_) of linear regression lines, *E*_*0*_ and *E*_*d*_ values are estimated and given in Tables 5, 6. Additionally, various optical parameters such as the static refractive index (*n*_*0*_), static dielectric constant (*ε*_*s*_) and the interaction strength between the material and electromagnetic radiation (*f*) are estimated and summarized in Table 4 using the following eqns.^56^:

18 $$n_{0} = \,\left( {1 + \frac{{E_{d} }}{{E_{0} }}} \right)^{1/2}, \;\;\varepsilon_{s} = n_{0}^{2}, \,\,f = E_{0} E_{d}$$

**Table 4:** Dispersion parameters of PVA/Cs/Ag nanocomposites.

Parameter	PVA/Cs blend	PVA/Cs/ 0.1wt%AgNPs	PVA/Cs/ 0.5wt%AgNPs	PVA/Cs/ 1.0wt%AgNPs
*E*_*0*_ (eV)	6.47	3.13	2.79	3.61
*E*_*d*_ (eV)	8.87	8.18	12.38	18.44
*n*_*0*_	1.73	1.90	2.33	2.47
*ε*_s_	2.99	3.61	5.44	6.10
*f* (eV)^2^	57.38	25.64	34.48	66.66
*M*_*-1*_	1.170	1.615	2.108	2.258
*M*_*-3*_, (eV)^−2^	0.027	0.164	0.271	0.172
*s*_*0*_ (×10^13^m^−2^)	1.202	1.065	1.519	0.769
*λ*_*0*_ (×10^−7^m)	3.623	3.559	3.317	3.920
*η*_*opt*_	1.960	1.91	1.82	1.793
*ε*_L_	4.82	8.55	8.06	8.00
N/m*(×10^58^m^−3^ kg^−1^)	0.47	1.05	0.18	0.14
*N* (×10^27^m^−3^)	1.89	4.20	0.72	0.56
*ω*_p_ (×10^15^ Hz)	3.69	5.51	2.28	2.02
τ (×10^–13^ s)	0.28	1.44	1.44	0.51
Μ (m^2^/s V)	0.011	0.057	0.057	0.021
P (×10^–7^ Ω m)	2.93	0.26	1.50	5.39
*χ*^(1)^	0.109	0.208	0.354	0.406
*χ*^(3)^ (×10^–12^ esu)	0.025	0.334	2.806	4.874
n_2_ (×10^–12^ esu)	0.621	1.906	2.167	2.413

It is found that these optical parameters are enhanced and increased with increasing the content of AgNPs. The dispersion energy (*E*_*d*_) values are increased from 8.87 for PVA/Cs blend to 18.44 for PVA/Cs/1wt% Ag nanocomposite sample. The improvement of these optical parameters with the increase of AgNPs content indicates that the electronic structure of the PVA/C matrix has undergone modification, and thus the defects has developed in the matrix of PVA/Cs blend. The first (*M*_*−1*_) and third (*M*_*−3*_) orders of the transition moments of the optical spectra that are related to the effective number of valence electrons in the samples are estimated as follow^57^:and

19 $$E_{0}^{2} = \,\frac{{M_{ - 1} \,}}{{M_{ - 3} }} \;\; \text{and} \;\; E_d^2 = \frac{{{M^3}_{ - 1}}}{{{M_{ - 3}}}}$$

It is noted that the values of *M*_*−1*_ and *M*_*−3*_ are increased upon increasing the AgNPs content. The Sellmeier oscillator classical model is used for further analysis of the refractive index dispersion to evaluate average oscillator wavelength (*λ*_*0*_) and average oscillator strength (*s*_*0*_)^58^. So, Wemple-Didomenico formula has been modified for as follows:

$$\frac{{n_{0}^{2} \, - \,1}}{{n^{2} \, - 1_{{}} }}\, = 1\, - \,\,\,\left( {\frac{{\lambda {}_{0}}}{\lambda }} \right)^{2}$$

20 $$(n^{2} \, - 1)^{ - 1} \, = \,\frac{{\lambda^{2} \, - \,\lambda_{0}^{2} }}{{(n_{0}^{2} \, - \,1)\,\lambda^{2} }}\, = \,\frac{1}{{(n_{0}^{2} \, - \,1)\,}}\, - \,\frac{{\,\lambda_{0}^{2} }}{{(n_{0}^{2} \, - \,1)\,}}\,\frac{1}{{\lambda^{2} }}$$

The dispersion Eq. (17) can be rewritten as follows in terms of λ:

21 $$\left( {n^{2} - 1} \right)^{ - 1} = \,\frac{{E_{0} \,}}{{E_{d} }} - \frac{{\,\left( {hc} \right)^{2} }}{{E_{o} E{}_{d}}}\frac{1}{{\lambda^{2} }}, \;\;E = \frac{hc}{\lambda }$$

Hence, from Eqs. (20) and (21), we get:and

22 $$\frac{{E_{0} }}{{E_{d} }}\, = \,\frac{1}{{n_{0}^{2} \, - \,1}} \;\; \text{and} \;\; \frac{{h^{2} c^{2} }}{{E_{0}^{{}} E_{d} }}\, = \,\frac{{\lambda_{0}^{2} }}{{n_{0}^{2} \, - \,1}}$$

Thus, we can obtain from Eq. (22):and

23 $$E_{0} \, = \,\,\frac{hc}{{\lambda_{0} }} \;\; \text{and} \;\; E_{d} \, = \,\,\frac{{hc\,(n_{0}^{2} \, - \,1)}}{{\lambda_{0} }}$$

Consequently, Eq. (21) can be rewritten in terms of (*λ*_*0*_) and (*s*_*0*_) as follow:

24 $$n^{2} - 1 = \,\,\frac{{\,s_{0} \lambda_{0}^{2} \,\lambda^{2} }}{{\lambda^{2} \, - \,\lambda_{0}^{2} }} \Rightarrow \left( {n^{2} - 1} \right)^{ - 1} = \,\frac{1}{{s_{0} \lambda_{0}^{2} }} - \,\,\frac{1}{{s_{0} }}\,\frac{1}{{\lambda^{2} }}$$

Figure 6c illustrates the dependence of (n^2^−1)^−1^ on λ^−2^ for PVA/Cs/Ag nanocomposite samples. Values of (*s*_*o*_) and (*λ*_*o*_) are estimated by the intercept and slope of the fitted curves of Fig. 6c and represented in Table 4.

According to the Drude model, the real and imaginary parts (ε_r_&ε_i_) of the dielectric constant depend on the wavelength of the incident photon as follows:

25 $$n^{2} = \varepsilon_{r} = \,\varepsilon_{L} \, - \,\frac{{e^{2} }}{{4\pi \varepsilon_{0} c^{2} }}\,\,\left( {\frac{N}{{m^{*} }}} \right)\,\,\lambda^{2}$$

26 $$\varepsilon_{i} = \,\,\frac{1}{{4\pi^{3} \varepsilon_{0} }}\frac{{e^{2} }}{{c^{3} }}\,\,\left( {\frac{N}{{m^{*} }}} \right)\,\,\left( {\frac{1}{\tau }} \right)\,\lambda^{3}$$

where *ε*_*L*_ is the lattice dielectric constant, *e* is the electric charge,* c* is light speed, *N* is the charge carrier concentration, m^*^ is the effective mass of the electron (m^*^ = 0.44 *m*_*e*_) and τ is the relaxation time, respectively. Figure 6d shows the variation of *n*^*2*^ against *λ*^*2*^ and values of (*ε*_*L*_) and (N/m^*^) are evaluated from the intercept and slope of the fitted lines of Fig. 6d, and demonstrated in Table 4. It is noted that *ε*_*L*_ are higher than *ε*_*s*_ and this difference is attributed to the contribution of the polarization that arises in the sample when exposed to light^8^. Furthermore, the values of plasma resonance frequency (*ω*_*p*_) are determined for all samples and summarized in Tables 5, 6 as follows:

27 $$\omega_{p} = \sqrt {\frac{{e^{2} }}{{\varepsilon_{0} }}\left( {\frac{N}{{m^{*} }}} \right)}$$

**Table 5:** The antimicrobial activity of PVA/Cs nanocomposites against various test microbes representing G + ve bacteria (*S. aureus*), G-ve bacteria (*E. coli*), Yeast (*C. albicans*) and fungi (*A. niger*).

Clear zone (ϕ mm)				Samples	
*A.niger*	*C. albicans*	*E. coli*	*S. aureus*	Samples name	Abbrev
0	0	0	0	PVA	A
0	0	0	0	Cs	B
0	0	0	0	PVA/Cs blend	C
0	0	0	0	PVA/Cs/0.1 wt%AgNPs	D
0	12	11	13	PVA/Cs/0.5 wt%AgNPs	E
0	15	14	16	PVA/Cs/1 wt%AgNPs	F

**Table 6:** The CFU value against and* S. aureus* and *E. coli.*

Bacteria	Sample	CFU at 10^–3^	
		CFU	R (%)
*S. aureus*	Control	84	–
	PVA	76	9.52
	Cs	65	22.62
	C (PVA/Cs)	45	46.43
	D	20	76.19
	E	4	95.24
	F	0	100.00
*E. coli*	Control	37	–
	PVA	23	37.84
	Cs	24	35.14
	C	15	59.46
	D	8	78.38
	E	4	94.59
	F	0	100.00

The dependence of the imaginary part (*ε*_*i*_) on the wavelength for PVA/Cs/Ag nanocomposites is displayed in Fig. 6e. From the slope values of the fitted lines according to Eq. (26), the relaxation time (τ) values are calculated and summarized in Table 4. Also, both the optical mobility (*μ*_*opt*_) and optical resistivity (*ρ*_*opt*_) are calculated and summarized in Table 4 by the following eqns.^59^:

28 $$\mu_{opt} = \frac{e\tau }{{m^{*} }} \;\; \text{and} \;\; \rho_{opt} = \frac{1}{{e\,N\,\mu_{opt} }}$$

### The optical electronegativity

The optical electronegativity (*η*_*opt*_) can be used to evaluate various physico-chemical parameters of the material^60^. The optical electronegativity refers to the tendency of the atom to attract electron to form ionic bonds. Duffy model is more accurate for the compositions used in optoelectronic structures and can therefore be used to calculate (*η*_*opt*_) values for PVA/Cs/Ag nanocomposites^61^. The values of (*η*_*opt*_) can be determined by the following equation^62^:

29 $$\eta_{opt} = \left( {\frac{Z}{{n_{o} }}} \right)^{1/4}$$

where Z is a constant equals to 25.54 for almost all materials^62^. *η*_*opt*_ values are calculated and given in Table 4. It is observed that, *η*_*opt*_ values are decreased from 1.960 to 1.793 as the content of AgNPs is increased. The decreasing of *η*_*opt*_ is attributed to its dependent on the refractive index, which is increased with increasing AgNPs content. The magnitude of *η*_*opt*_ refers to the bonding nature in the material. The small values of *η*_*opt*_ and the relatively high refractive index values of PVA/Cs/Ag nanocomposites may attributed to their covalent nature^62^.

### Linear/nonlinear optical Properties

The nonlinear optical properties have an important role in the fabrication of many nonlinear optical devices such as integrated photonic devices and all-optical switching devices. The source of optical nonlinearity in the material arises when the bond lengths are affected by the nuclear interaction with the electronic polarization^63^. When an electromagnetic wave passes through the material, the dipole orientation takes place leading to a non-linear electronic polarizability (P) because of their interactions with charged particles. The density of polarization (P) is correlated directly to the susceptibility (χ), i.e., *P* = *ε*_*0*_* χ E*, where *E* is the intensity of the electric field strength. The susceptibility (*χ*) equals to the sum of both linear (*χ*^L^) and nonlinear contribution (*χ*^NL^):

30 $$\chi = \chi^{L} \, + \,\chi^{NL}$$

where *χ*^L^ refers to the linear part of susceptibility (*χ*^(1)^) while *χ*^NL^ describes the nonlinear parts of susceptibility (*χ*^(2)^ , *χ*^(3)^). So, the total susceptibility can be expressed as follows:

31 $$\chi = \chi^{(1)} \, + \,\chi^{(2)} \, + \chi^{(3)}$$

The first and third-order optical susceptibilities can be represented in terms of linear refractive index by the following equation^64^:

32 $$\chi^{(1)} = \,\,\left( {n_{0}^{2} \, - 1} \right)/4\pi$$

And

$$\chi^{(3)} = \,\,1.79x10^{ - 10} \,x\,\left( {\chi^{(1)} } \right)^{4}$$

33 $$\chi^{(3)} = \,\,\frac{{1.79x10^{ - 10} }}{{\left( {4\pi } \right)^{4} }}\,\left( {n_{0}^{2} \, - 1} \right)^{4}$$

The nonlinear refractive index (*n*_*2*_) also can be represented in terms of *χ*^*(3)*^ as follow^64^:

34 $$n{}_{2} = \,\,\frac{{12\,\pi \,\chi^{(3)} }}{{n_{0} }}$$

Linear/nonlinear optical parameters are calculated for our investigated samples and listed in Table 4. It is noted that these parameters are enhanced and increased upon increasing the content of AgNPs. This enhancement can be related to the existence of defect centers because of the interaction between AgNPs and PVA/Cs chains, resulting in an increase in the local polarization. Such control in the values of these optical properties through the incorporation AgNPs makes PVA/Cs/Ag nanocomposites are suitable for various modern photonic applications like high-speed communication devices and ultra-fast optical switching^64^.

The nonlinear absorption coefficient (β_c_) is estimated using the following equation^65^:

35 $$\beta {}_{c}\, = \,\frac{{48\,\pi^{3} \,\chi^{(3)} }}{{c\,\lambda \,n^{2} }}$$

**Fig. 7:**
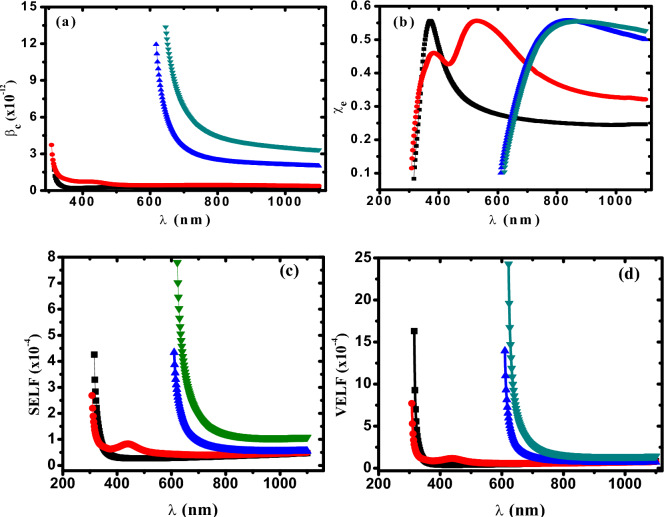
(**a**) β_c_ versus λ, (**b**) χ_e_ versus λ, (**c**) SELF versus λ and (**d**) VELF versus λ (Filled black square) PVC/Cs, (filled red circle) PVC/Cs/0.1 wt% AgNPs, (filled blue triangle) PVC/Cs/0.5 wt% AgNPs (filled green inverted triangle) PVC/Cs/1 wt% AgNPs.

Figure 7a displays the dependence of nonlinear absorption coefficient β_c_ on the wavelength for PVA/Cs/Ag nanocomposites.

It is clear that β_c_ has high values at lower wavelength (high photon energy) and is gradually decreased with increasing the wavelength. Moreover, it is found that the β_c_ increases with increasing the content of AgNP due to the increased number of excited electrons overcoming the bandgap. The electrical susceptibility (*χ*_*e*_) of the investigated samples is calculated by the following equation^66^:

36 $$\chi_{e} \, = \,\frac{{\left( {n^{2} \, - \,k^{2} \, - \,\varepsilon_{0} } \right)}}{4\,\pi }$$

Figure 7b illustrates the dependence of electrical susceptibility (*χ*_*e*_) on the wavelength. It is observed that *χ*_*e*_ increases with increasing wavelength and then begins to decrease at higher wavelengths. The *χ*_*e*_ also increased with increasing AgNPs content due to increased electron mobility as well as more defects, which led to an increase in the localized density of states.

### Surface and volume energy loss function (SELF&VELF)

Because of the excitation of plasma oscillations in the sea of conduction electrons, it is suggested that fast-moving electrons as they pass through the material may experience a characteristic energy loss. This energy loss is closely correlated with the optical properties, especially the dielectric constant of the material. The rapid loss of energy by electrons as they propagate through the surface of the material or through the bulk of the material is defined as a function of surface and volume energy loss. The optical transition of electrons in PVA/Cs/Ag nanocomposites is investigated using the surface and volume energy loss functions (SELF&VELF) that describe the energy loss of fast electrons within the material. SELF and VELF can be described in terms of the real part (*ε*_*r*_ = *n*^*2*^*-k*^*2*^) and imaginary part of dielectric constant (*ε*_*i*_ = *2nk*) using the following equations^67^:

37 $$SELF = \,\frac{{\varepsilon_{i}^{2} }}{{\left( {\varepsilon_{r} + 1} \right)^{2} \, + \,\varepsilon_{i}^{2} }}$$

38 $$VELF = \,\frac{{\varepsilon_{i}^{2} }}{{\varepsilon_{r}^{2} \, + \,\varepsilon_{i}^{2} }}$$

Figure 7c,d shows the variation of SELF and VELF for PVA/Cs/Ag nanocomposites versus wavelength. It is observed that SELF and VELF are decreased rapidly with increasing wavelength. Also, SELF and VELF are enhanced and increased with increasing the content of AgNPs in PVA/Cs matrix. Gernerally, it is observed that the energy lost through the surface of the material is lower than that lost in the bulk at any wavelength for all investigated samples and this behavior is attributed to different effects such as scattering and collision of electrons.

### Optical conductivity (σ_opt_) and dielectric constant

The photoconductivity (*σ*_*opt*_) and electrical conductivity (*σ*_*elec*_) describing the conductivity that is produced as a result of the mobility of charge carrier due to the electric field variation of the incident electromagnetic waves are estimated in terms of the refractive index and absorption coefficient, as follow^68^:

39 $$\sigma_{opt} = \,\,\frac{\alpha \,n\,c}{{4\,\pi }}$$

40 $$\sigma_{e} = \,\,\frac{{2\,\lambda \sigma_{opt} }}{\alpha }$$

**Fig. 8:**
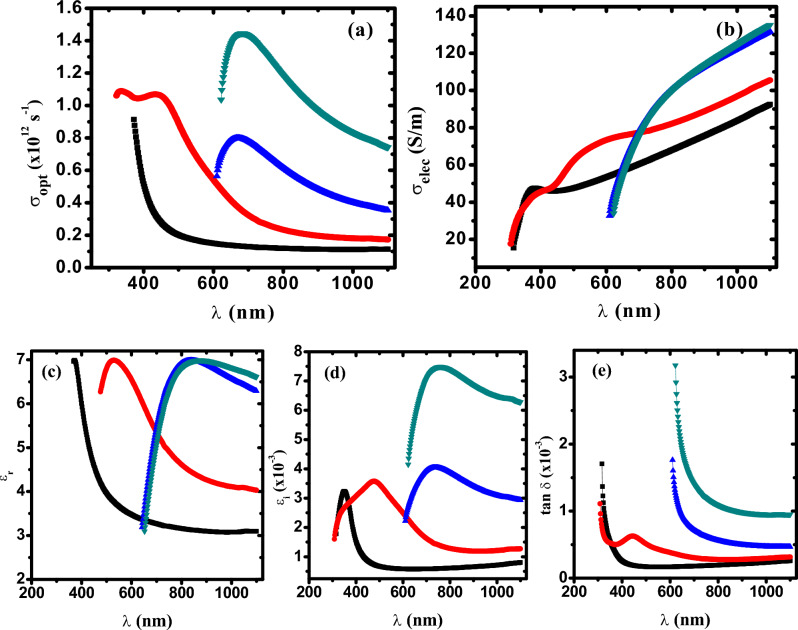
(**a**) σ_opt_ versus λ, (**b**) σ_elec_ versus λ, (**c**) ε_r_ versus λ, (**d**) ε_i_ versus λ and (**d**) tan δ versus λ. (Filled black square) PVC/Cs, (filled red circle) PVC/Cs/0.1 wt% AgNPs, (filled blue triangle) PVC/Cs/0.5 wt% AgNPs (filled green inverted triangle) PVC/Cs/1 wt% AgNPs.

Figure 8a,b displays the variation of optical and electrical conductivities of PVA/Cs/Ag nanocomposites against the wavelength. It is observed that the optical and electrical conductivities behaved in different ways as a function of the wavelength. The optical conductivity is found to be in the order of 10^12^ s^−1^ indicating that PVA/Cs/Ag nanocomposites have a high photo-response, making it strong candidate for using in the information processing. The high values of photoconductivity in the high absorption region i.e., at lower wavelength (higher energy) are attributed to the increased density of charge carriers which resulted from the increased content of silver nanoparticles. The reduction of the optical band gap with increasing AgNPs content in the nanocomposite samples due to the increase in the concentration of charge carriers as well as the number of localized energy states, led to an increase in the optical conductivity.

Moreover, on the other hand, it is found that the electrical conductivity value increased from 62 to ~ 135 S/m with increasing AgNPs content, indicating the semiconducting nature of the PVA/Cs/Ag nanocomposites. The decrease in the optical bandgap and enhancement of the optical conductivity (*σ*_*opt*_) with increasing the content of AgNPs in PVA/Cs matrix confirm that optical and dielectric measurements are in good agreement with each other. The optical conductivity and optical dielectric results reflect the importance of PVA/Cs/Ag nanocomposites in the design of optoelectronic devices.

The dielectric constant describes the material’s polarizability that depends mainly on density of states (DOS) in the energy gap. When a material is exposed to an electric field, the polarization of the atoms, ions, or molecules of the material greatly affects some optical constants such as (*n*,*k*) in the anisotropic medium. The polarization takes place within the material due to the reorientation of the center of negative charge relative to the positive one. When light propagate through a material, the accompanying electric field will polarize the atoms, ions or molecules, but the resulting polarization does not react quickly with the applied field. The complex optical dielectric constant can be expressed as follows:

41 $$\varepsilon^{*} = \varepsilon_{r} - \,i\,\varepsilon_{i}$$

The real and imaginary parts of optical dielectric constant are estimated by the following equations^5^:

42 $$\varepsilon_{r} = n^{2} - k^{2}$$

43 $$\varepsilon_{i} = 2nk$$

The dependence of the real and imaginary parts of dielectric constant on the wavelength for PVA/Cs/Ag nanocomposites is illustrated in Fig. 8c,d. It is found that *ε*_r_ and *ε*_i_ are nearly behaved with the same trend with increasing the content of AgNPs as well as the wavelength. Both *ε*_r_ and *ε*_i_ are found to be increased with increasing AgNPs content. Higher values of *ε*_r_ and *ε*_i_ in the lower wavelength (higher photon energy) region are attributed to the contribution of charge carriers and interfacial polarization^69–71^. Moreover, the behavior of *ε*_r_ and *ε*_i_ exhibited a wide region of dispersion because of the polar nature of PVA/Cs/Ag nanocomposites which follow the incident field fluctuation^5,26^.

Dielectric loss (tan δ = ε″/εʹ) is a widespread factor for evaluating the absorbed and dissipated energy of materials. The damping can be denoted by tan δ which is estimated from the ratio of loss to energy storage. Dielectric loss tan δ expresses the level of degradation in the manufactured composites. The variation of tan δ exhibited a sharp decrease with increasing the wavelength followed by almost constant values at high wavelengths, as shown in Fig. 8e. Also, it is observed that tan δ is increased with increasing the content of AgNPs in the matrix of PVA/Cs. Hence, we can say that the embedding of AgNPs in PVA/Cs matrix leads to the creation of new molecular dipole levels as point defects within the HOMO-LOMO gap and thus the dielectric loss is enhanced.

### Antibacterial activity

**Fig. 9:**
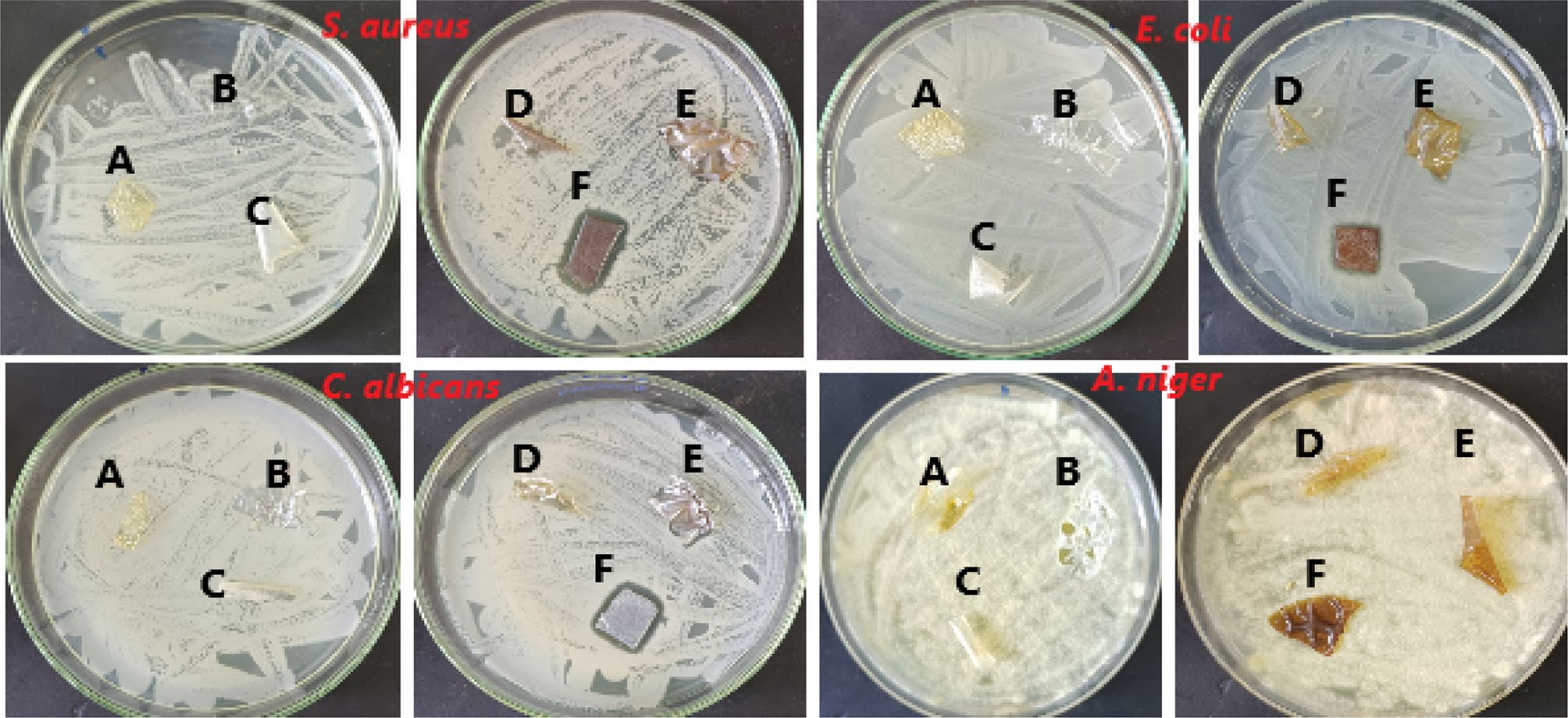
The bacterial inhibition zone of PVA, Cs and PVA/Cs/AgNPs.

The antimicrobial activity of the prepared samples is primarily tested using agar diffusion method. Results in Table 5 and Fig. 9 revealed that samples A, B, C and D didn’t exhibit any antimicrobial activity against all test microbes. Sample E showed low antimicrobial activity against *S. aureus*, *E. coli* and *C. albicans* whereas sample F showed promising antimicrobial against the same test microbes (*S. aureus*, *E. coli* and *C. albicans*). Both samples E and F didn’t have antifungal activity against *A. niger*.

**Fig. 10:**
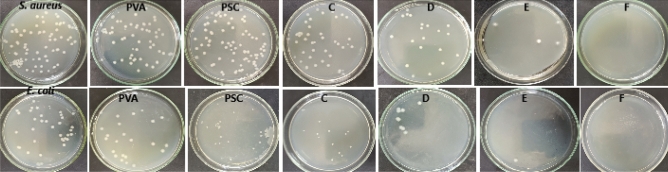
The CFU value against and* S. aureus* and *E. coli.*

The antibacterial activity of the prepared samples has been reinforced using the colony forming unit (CFU) method. Results in Table 6 and Fig. 10 showed that samples A and B showed low very reduction in growth against *S. aureus* (9.52 and 22.62%, respectively) and *E. coli* (37.84 and 35.14%, respectively). Samples C and D showed moderate antibacterial against *S. aureus* (46.43 and 76.19%, respectively) and *E. coli* (59.46 and 78.38%, respectively). Samples E and F showed potent growth reduction against *S. aureus* (95.24 and 100.00, respectively) and *E. coli* (94.59 and 100.00, respectively).

Our results are in good agreement with those previously published by Kharaghani et al.who studied the antibacterial activity of PVA/chitosan doped with CuNPs/AgNPs and with Souflet et al. who studied the antibacterial activity of chitosan/PVA hydrogel doped with AgNPs and also with Abdel Fattah et al.who studied the antibacterial activity of the PVA/chitosan doped with ZnONPs^5,31,72^. These studies demonstrated that these nanocomposites have excellent antibacterial activity, making them strong candidates for their many applications in medical fields.

Antibacterial agents are classified into two types: bactericidal agents, which kills bacteria; and antibacterial agents, which inhibit the growth of bacteria. It is known that the embedding of nanoparticles (NPs) into the biopolymer matrix leads to an increase in their antibacterial activity. The antibacterial activity mechanism depends mainly on the natural properties and species of bacteria as well as the type of NPs. The sensitivity of bacteria to nanoparticles is also affected by their rate of growth as well as the structure of the bacterial cell wall^73^. Currently, the literature mainly supports three mechanisms for the antibacterial effect of silver nanoparticles^74^. The first mechanism assumes that silver nanoparticles act at the membrane level due to their ability to penetrate the outer membrane, then accumulate in the inner membrane of the bacteria and the adhesion of AgNPs to the cell leads to its destabilization and then damage, which increases the permeability of the membrane and this in turn leads to the leakage of the cell content and thus its death^75^. It has also been shown that silver nanoparticles interact with proteins present in the cell wall of bacteria that contain sulfur and this interaction causes structural damage that ultimately leads to the rupture of the cell wall. The second mechanism suggests that AgNPs can penetrate the cell membrane, alter its permeability and structure, and then enter the cell. Silver nanoparticles, due to their properties, can interact with phosphorus or sulfur groups exist in the intracellular content such as proteins and DNA, altering their functions as well as their structure. Similarly, they may also change the respiratory chains in the inner membrane by interaction with thiol group in enzymes that catalyze the reactive oxygen species (ROS) and free radicals, damaging the intracellular machinery and accelerating cell death. The third mechanism is the release of silver ions from nanoparticles, which due to their charge and size will interact with cellular components and thus alter membranes, metabolic pathways and even the genetic material and is proposed to occur in parallel with the other two mechanisms.

## Conclusion

Analysis of XRD measurements showed that the structural parameters of PVA/Cs matrix have been enhanced with increasing the content of Ag nanoparticles. FT-IR analysis exhibited that the main characteristic bands of PVA/Cs blend is affected and showed blue and red shifts in the band position, indicating that the interaction between PVA/Cs chains and Ag nanoparticles are achieved. UV/Vis measurements exhibited that the values of E_U_ is increased from 1 eV for pure PVA/Cs blend to 2.38 eV for PVA/Cs/1 wt% Ag nanocomposite, and for the same samples, the direct and indirect band gap values ​​decrease from 4.34/5.22 eV to 3.38/4.78 eV. The reduction of the optical bandgap is interpreted based on the creation of defects and new localized states, as verified by the values of Urbach energy. Wemple-DiDomenico (WDD) model is applied to estimate the dispersion parameters and linear/nonlinear parameters of PVA/Cs/Ag nanocomposites. Values of *ε*_*s*_ and *ε*_*L*_ are increased from 2.99/4.82 for pure PVA/Cs blend to 6.10/8 for PVA/Cs/1 wt% Ag nanocomposite. A clear enrichment in the linear/nonlinear optical parameters is observed upon increasing AgNPs content. Also, it is found that the real (ε_r_) and imaginary (ε_i_) parts of the complex dielectric constant and tan δ are decreased with increasing the wavelength. The novel findings in this work nominate PVA/Cs/Ag nanocomposites for linear/nonlinear optical devices applications. Also, the antibacterial activity of PVA/Cs/Ag nanocomposite samples is investigated against various Gram-positive and Gram-negative bacteria**.** The antibacterial activity of these nanocomposites recommends its use in the fabrication of antibacterial devices with the biomedical application**.**

## Data Availability

Datasets generated during the current study are available from the corresponding author on reasonable request.
